# Functional analysis of the Landsberg *erecta* allele of *FRIGIDA*

**DOI:** 10.1186/s12870-014-0218-2

**Published:** 2014-08-13

**Authors:** Inga Schmalenbach, Lei Zhang, Malgorzata Ryngajllo, José M Jiménez-Gómez

**Affiliations:** Department of Plant Breeding and Genetics, Max Planck Institute for Plant Breeding Research, Carl-von-Linné-Weg 10, 50829 Cologne, Germany; INRA, UMR1318, Institut Jean-Pierre Bourgin, RD10, F-78000 Versailles, France

**Keywords:** *Arabidopsis thaliana*, Flowering time, *FRIGIDA*, Vernalization, Natural variation, Allelic series

## Abstract

**Background:**

Most of the natural variation in flowering time in *Arabidopsis thaliana* can be attributed to allelic variation at the gene *FRIGIDA* (*FRI,* AT4G00650), which activates expression of the floral repressor *FLOWERING LOCUS C* (*FLC*, AT5G10140). Usually, late-flowering accessions carry functional *FRI* alleles (*FRI-wt*), whereas early flowering accessions contain non-functional alleles. The two most frequent alleles found in early flowering accessions are the ones present in the commonly used lab strains Columbia (*FRI-Col*) and Landsberg *erecta* (*FRI-Ler*), which contain a premature stop codon and a deletion of the start codon respectively.

**Results:**

Analysis of flowering time data from various Arabidopsis natural accessions indicated that the *FRI-Ler* allele retains some functionality. We generated transgenic lines carrying the *FRI-Col* or *FRI-Ler* allele in order to compare their effect on flowering time, vernalization response and *FLC* expression in the same genetic background. We characterize their modes of regulation through allele-specific expression and their relevance in nature through re-analysis of published datasets. We demonstrate that the *FRI-Ler* allele induces *FLC* expression, delays flowering time and confers sensitivity to vernalization in contrast to the true null *FRI-Col* allele. Nevertheless, the *FRI-Ler* allele revealed a weaker effect when compared to the fully functional *FRI-wt* allele, mainly due to reduced expression.

**Conclusions:**

The present study defines for the first time the existence of a new class of Arabidopsis accessions with an intermediate phenotype between slow and rapid cycling types. Although using available data from a common garden experiment we cannot observe fitness differences between accessions carrying the *FRI-Ler* or the *FRI-Col* allele, the phenotypic changes observed in the lab suggest that variation in these alleles could play a role in adaptation to specific natural environments.

**Electronic supplementary material:**

The online version of this article (doi:10.1186/s12870-014-0218-2) contains supplementary material, which is available to authorized users.

## Background

As plants are sessile organisms, adaptation to the environment is essential for their survival and reproductive success. Mechanisms regulating the response to environmental cues enable a proper timing of key events in a plant’s life. One crucial event, resulting from the integration of endogenous and environmental signals, is the switch from vegetative to reproductive development. The annual species *Arabidopsis thaliana* occurs in the northern hemisphere in a broad range of latitudes differing substantially in day length, temperature and other ecological factors [1]. As a result of adaptation to specific habitats, Arabidopsis accessions have evolved two main life history strategies. Winter-annuals germinate in autumn, survive winter as a rosette and flower in the following summer, whereas summer-annuals germinate in spring or summer and finish their reproduction cycle in the same year. Variation between these distinct strategies has been associated with allelic variation at the genes *FRIGIDA* (*FRI*) and *FLOWERING LOCUS C* (*FLC*), which act epistatically to regulate flowering time [2,3]. Arabidopsis individuals containing functional alleles at these two loci flower very late or not at all, unless they receive a prolonged exposure to cold (vernalization).

*FLC* encodes a MADS-box transcription factor that binds to the promoters of floral initiators such as *FLOWERING LOCUS T (FT)* and *SUPPRESSOR OF CONSTANS OVEREXPRESSION 1 (SOC1)* to repress their transcription. Flowering occurs when *FLC* is downregulated by proteins of the vernalization and/or autonomous pathways, reviewed in [4-7]. Transcriptional activation of *FLC* requires integrated activity of diverse chromatin remodeling and histone-modifying complexes, reviewed in [8]. When Arabidopsis plants are vernalized, expression of *FLC* is decreased and maintained at reduced level by different epigenetic marks in a Polycomb-mediated process involving long non-coding RNAs [9-11]. The epigenetic silencing of *FLC* is quantitatively modulated and underlies Arabidopsis natural variation for vernalization response [12-14]. Two main haplogroups of *FLC* have been defined mainly by polymorphisms within the first intron, an important region to maintain silencing induced by vernalization [15,16]. These haplogroups were shown to underlie differences in flowering time among natural accessions of Arabidopsis, but only when a functional *FRI* allele is present [15].

Despite the central role of *FLC*, most of the variation in flowering time has been found to correlate with natural allelic diversity at *FRI* [3]. *FRI* is the founding member of a family of seven Arabidopsis proteins that contain two coiled-coil domains and show no homology to other proteins [2,17]. In order to regulate flowering time, *FRI* builds the scaffold protein of a transcription activator complex that mediates diverse chromatin modifications at *FLC* [18,19]. Furthermore, *FRI* is suggested to be involved in co-transcriptional processes that link the function of 5′ end capping with transcription and efficient splicing of *FLC* [20]. Vernalization abolishes the effect of *FRI* and silences *FLC* as described above [9,12].

A considerable number of different *FRI* haplotypes have been identified within accessions from a wide range of latitudes [14,21,22] or more restricted geographic regions [23,24]. Studies on these alleles led to the conclusion that early flowering types evolved by multiple independent mutational events from winter-annuals containing an ancestral functional *FRI* allele (*FRI-wt*) [25]. Two distinct deletions in *FRI* are believed to confer early flowering in most of the rapid cycling accessions. The Columbia allele (*FRI-Col*) carries a 16 bp-deletion resulting in a premature stop codon and, thus, a truncated protein missing a part of the C-terminal [2,14]. But the most frequent deleterious *FRI* mutation in nature is a 376 bp-deletion combined with a 31 bp-insertion in the promoter as observed in Landsberg *erecta* (*FRI-Ler*) [14,21,22]. This mutation disrupts the translational start but, due to a second alternative start codon, a short out-of-frame protein might be built [2]. The loss-of-function *FRI* alleles found in L*er* and Col are widely used as examples of positive selection towards rapid cycling accessions [26,27].

Although the *FRI-Ler* and *FRI-Col* alleles are always classified as a single non-functional group, there are evidences for differences in their effects. First, accessions carrying the L*er-*type deletion but not the Col-type deletion show considerable variation in flowering time and *FLC* levels [14,28]. Then, several studies find variation in flowering time associated with the chromosomal region of *FRI* in mapping populations derived from accessions containing the *FRI-Ler* allele crossed to accessions with a loss-of-function allele [28-30]. In all these cases, the L*er* allele was associated with a delay in flowering time. This delay was attributed to additional loci in the same region as *FRI*, although these have never been identified.

This article provides the first robust evidence that the L*er* allele of *FRI* is functional. In contrast to the true null *FRI-Col* allele, the *FRI-Ler* allele is able to induce *FLC* expression, resulting in delayed flowering and increased vernalization sensitivity. Nevertheless, the *FRI-Ler* allele has a weaker effect than the fully functional *FRI-wt* allele. The reduced functionality of the *FRI-Ler* allele mainly results from its lower expression putatively due to cis regulatory polymorphisms in its promoter. Our finding defines a new functional class of *FRI* alleles, in which accessions carrying the *FRI-Ler* allele would flower in between the late and early flowering groups defined so far.

## Results

### Effects of the *FRI-Ler* and *FRI-Col* alleles on flowering time and vernalization response

**Figure 1:**
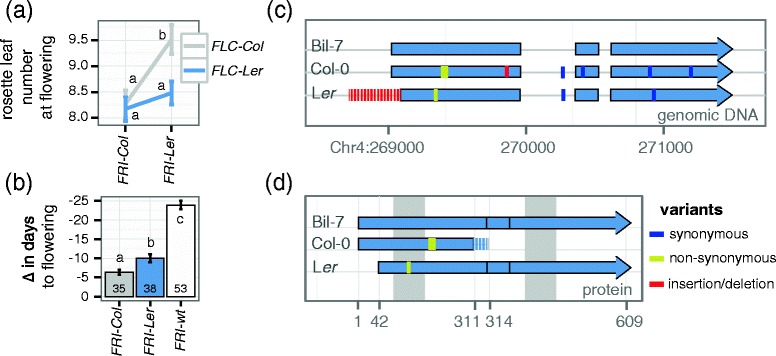
**Functional comparison of the most common deleterious** ***FRI*** **alleles. (a)** Leaf number at flowering from individuals in the Col x L*er* recombinant inbred line set grouped by their genotype at *FRI* and *FLC*. **(b)** Vernalization response of Arabidopsis natural accessions measured as the reduction of days to flowering in plants vernalized for 5 weeks at 4°C compared to unvernalized plants. Accessions are grouped by their *FRI* alleles as described in [22]. The number at the bottom of each bar indicates the number of accessions in each group; error bars in **(a)** and **(b)** correspond to the standard error of the mean. Letters in each bar or point represent significance groups as determined by Tukey HSD test. **(c)** Exonic organization for *FRI-wt* (Bil-7), *FRI-Col* and *FRI-Ler.* Red regions indicate the indels characteristic for *FRI-Col* and *FRI-Ler*. **(d)** Predicted proteins for all three alleles shown in **(c)**. The dashed region in *FRI-Col* represents a shift in the ORF caused by the 16 bp deletion. Shaded regions in the background indicate predicted coiled-coil domains.

We investigated if the *FRI* alleles present in Col-0 and L*er* show evidences of different functionality by analyzing flowering time data from a recombinant inbred line (RIL) population derived from a cross between the two accessions [31]. Because the effect of *FRI* on flowering time depends on the alleles at *FLC*, we took into account the genotype of the RILs at both loci [15]. While Col-0 harbors a functional *FLC* allele, L*er* has been described to contain a weak allele with a transposon-like insertion in the first intron [25,32,33]. As shown in Figure 1a, plants carrying the *FRI-Ler* allele flower significantly later than plants carrying the *FRI-Col* allele, although only in the presence of the strong *FLC* allele of Col-0 (two-way ANOVA, *FRI* p = 0.0095, *FLC* p = 0.0138, interaction p = 0.0824). This epistatic interaction suggests the existence of a functional *FRI* allele in L*er*, as no other gene located in this region of chromosome 4 has been shown to delay flowering time through interactions with a locus in the region of chromosome 5 containing *FLC*.

Because strong vernalization requirements are associated with functional *FRI* alleles, we decided to analyze variation in vernalization sensitivity among Arabidopsis accessions [34,35]. We calculated the decrease in flowering time in response to vernalization in a published dataset containing 126 Arabidopsis accessions classified as *FRI-Col, FRI-Ler* or *FRI-wt* [22]. Accessions carrying the *FRI-Ler* allele accelerated flowering by 10 days in response to vernalization (Figure 1b). This acceleration was intermediate to the low response of accessions containing the *FRI-Col* deletion and the high response of accessions carrying the *FRI-wt* allele (Figure 1b). The same trend was found for the vernalization response quantified as total leaf number, although the difference between accessions carrying the *FRI-Ler* allele and the *FRI-Col* allele was not statistically significant (Additional file 1).

Taken together, our results disagree with the common belief that the *FRI* allele present in L*er* is not functional. However, from this analysis we cannot completely rule out the possibility that the observed phenotypes are caused by additional loci in linkage disequilibrium with *FRI*.

### Structural characterization of the *FRI-Ler* and *FRI-Col* alleles

**Figure 2:**
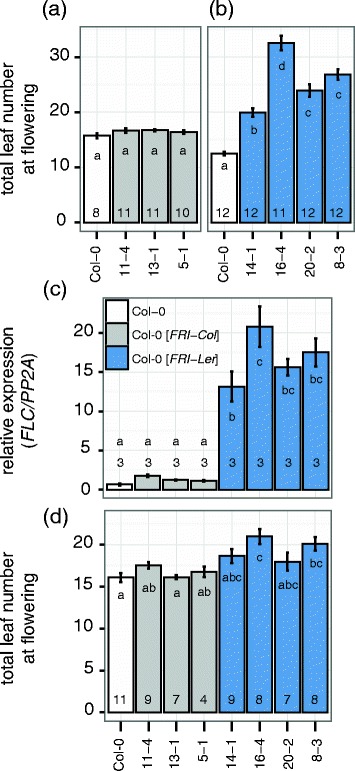
**Characterization of transgenic lines carrying the** ***FRI-Col*** **or** ***FRI-Ler*** **allele. (a)** Flowering time expressed as total leaf number (rosette + cauline) from homozygous T_3_ single insertion lines containing a *FRI-Col* transgene are shown in comparison to the untransformed Col-0 wild type. **(b)** Experiment as in **(a)** but using homozygous T_3_ single insertion lines transformed with *FRI-Ler*. **(c)** Expression of *FLC* for the same genotypes as above. In this experiment, leaf tissue was collected from 10 day-old seedlings grown in long day conditions. The number in each bar indicates the number of biological replicates used. Expression was normalized to the expression of *PP2A*. **(d)** Flowering time after vernalization quantified as total leaf number for the same lines as above. Plants were vernalized for four weeks at 4°C and subsequently grown under long day conditions. The number in each bar indicates the number of individual plants per line analyzed. Error bars represent the standard error of the mean. Letters in each bar represent significance groups as determined by Tukey HSD test.

We looked for further evidences of a functional *FRI-Ler* allele by studying sequence variation among Arabidopsis accessions. We sequenced genomic DNA from *FRI* including its upstream and downstream regions in Col-0, L*er*-1 and Bil-7, the latter being a winter-annual accession that contains a functional *FRI-wt* allele identical to the published H51 allele [2,14]. All polymorphisms found in *FRI-Col* and *FRI-Ler* correspond exactly to the ones described before, such as the insertion/deletions that define these two allelic classes (Figure 1c; [2,14]). The H51 allele is predicted to encode a fully functional 609 aa protein [GenBank: AAG23415] while the Col allele yields a 314 aa protein [GenBank: AEE81913], truncated due to a premature stop codon resulting from a 16 bp deletion in exon 1 (Figure 1c and d; [2]). In the case of *FRI-Ler*, a 376 bp deletion combined with a 31 bp insertion removes the translational start, but creates a new, out-of-frame start codon that is predicted to yield a 41 aa protein [2]. Interestingly, in addition to the out-of-frame start codon, *FRI-Ler* contains an in-frame ATG codon downstream of the original start, which would result in a protein missing 42 aa of the N-terminus. Apart from this deletion and one conservative amino acid change from L to I in the first coiled-coil domain, the FRI-L*er* protein is identical to the functional Bil-7 allele (Figure 1d). There are no known functional motifs in the deleted segment of the FRI protein, and, thus, if transcription and translation occurred from the downstream in-frame start codon, the resulting FRI-L*er* protein could possibly be as functional as the full-length Bil-7 protein.

We looked for evidences of transcription of this long *FRI-Ler* allele using RNA-seq reads from seven accessions containing the characteristic L*er* indel [36]. We found signals of expression across the full length of the gene, including the C-terminal part, in all accessions (see Additional file 2). This raised the question of whether this long *FRI-Ler* transcript is translated into a protein and if so, whether it is sufficient to delay flowering and to confer vernalization sensitivity.

### Comparing the effect of the *FRI-Ler* versus the *FRI-Col* allele in transgenics

We studied the existence of a functional *FRI-Ler* allele by comparing its function with that of the *FRI-Col* allele in transgenic plants. For this, we cloned the putative coding region plus the upstream and downstream region of both alleles and transformed them in a common background. We chose Col-0 as a recipient because our previous results suggest that *FRI-Col* carries a loss-of-function mutation (Figure 1), and because Col-0 has been shown to contain a strong *FLC* allele that is activated in the presence of a *FRI-wt* allele [3]. First, we confirmed the lack of function of the *FRI-Col* allele by growing three independent T_3_ lines homozygous for the *FRI-Col* transgene (Col-0[*FRI-Col*]) under long day conditions in the greenhouse. In this experiment none of the transgenic lines was significantly different from wild type (Figure 2a). In a consecutive experiment in similar conditions we grew homozygous T_3_ transgenic lines containing the *FRI-Ler* transgene (Col-0[*FRI-Ler*]) and observed a significant delay in flowering in all lines compared with the Col-0 wild type (Figure 2b), confirming *FRI-Ler* functionality.

Because *FRI* delays flowering time through upregulation of the floral repressor *FLC* [3], we performed qRT-PCR to test the expression of *FLC* in the transgenic plants. As expected, we found elevated expression of *FLC* associated with the *FRI-Ler* transgene but not with the *FRI*-Col transgene (Figure 2c), despite all lines presenting increased *FRI* expression (Additional file 3). In addition, a prolonged cold treatment, known to abolish *FLC* expression, significantly reduced leaf number in Col-0[*FRI-Ler*] lines, but not in Col-0 wild type or in Col-0[*FRI-Col*] lines (p < 0.001, p = 0.06 and p = 0.28 for the interaction between vernalization and genotype in a two-way ANOVA respectively, compare Figure 2d and Figure 2a and b).

Altogether, our results demonstrate that the *FRI-Ler* allele, but not the *FRI-Col* allele, has the ability to delay flowering*,* and that this delay is achieved through upregulation of *FLC*.

### Expression of *FRI* and *FLC* in natural accessions

The functionality of the *FRI-Ler* allele is contrasting with its frequent presence among early flowering accessions [2,14]. Our analyses also show that, although accessions carrying *FRI-Ler* alleles have stronger vernalization responses than accessions containing *FRI-Col* alleles, they do not reach the levels observed in lines carrying *FRI-wt* alleles (Figure 1b and Additional file 1). This suggests a reduced functionality of the *FRI-Ler* allele in comparison with the *FRI-wt* allele, which can be caused by differences in the protein sequence, in expression levels or in post-transcriptional/post-translational mechanisms. Previous works have pointed to the low expression of the *FRI-Ler* allele, possibly as a consequence of the indel in its promoter [2,37]. This led us to focus on a possible transcription inhibition as the cause for early flowering in natural accessions containing this allele.

**Figure 3:**
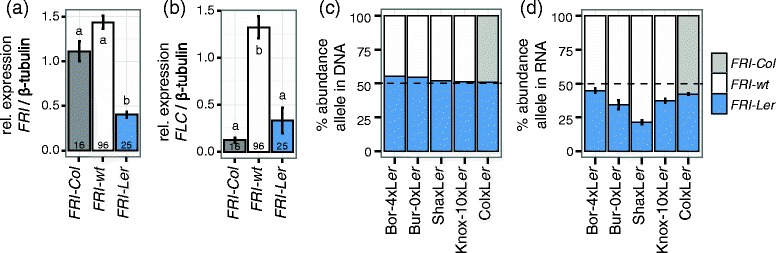
**Relative expression levels of** ***FRI*** **and** ***FLC*** **in Arabidopsis accessions and F**_**1**_ **hybrids grouped by their** ***FRI*** **allele. (a and b)** 137 Arabidopsis accessions were grown in long days under greenhouse conditions, sampled after four weeks and expression levels of *FLC* and *FRI* were quantified from northern hybridizations [38]. Letters in each bar indicate the significance groups as determined by a Tukey HSD test. The number in each bar indicates the number of accessions in each group. **(c and d)** Positions with SNPs specific to L*er*-1 or Col-0 were targeted using pyrosequencing in genomic DNA **(c)** or in cDNA **(d)** of F_1_ hybrids. Leaf material for hybrids involving Bor-4, Bur-0, Knox-10 or Sha was collected at the time of bolting from plants growing under 12-hour photoperiods in an environmental chamber. Leaf material from hybrids involving Col-0 was collected from 10 day-old seedlings grown in the greenhouse in long days. Error bars indicate the standard error of the mean.

We analyzed the expression profiles of *FRI* and its downstream target *FLC* in 137 Arabidopsis accessions containing *FRI-Ler*, *FRI-Col* or *FRI-wt* alleles [38]. Accessions carrying *FRI-Ler* alleles showed significantly reduced expression of *FRI* when compared to accessions carrying *FRI-Col* or *FRI-wt* alleles, suggesting a regulatory defect in the *FRI-Ler* allele (Figure 3a). Interestingly, this residual *FRI-Ler* expression results in higher *FLC* levels than in the accessions carrying *FRI-Col* alleles, although this difference is not significant (Tukey HSD test p value p = 0.079), possibly due to the large variation in *FLC* expression found among accessions with *FRI-Ler* alleles (compare error bars in Figure 3b).

### Allele-specific expression of *FRI-Ler* and *FRI-Col*

There are two alternative causes for the lower *FRI* expression found in Arabidopsis accessions carrying the *FRI-Ler* allele when compared to accessions carrying *FRI-wt* or *FRI-Col* alleles. Either the *FRI*-L*er* allele contains a mutation in cis, such as the promoter indel that defines the class, or its reduced expression is caused by the effect of trans-regulators that may occur in the early flowering accessions. In order to distinguish among these possibilities, we analyzed the allele specific expression of *FRI* using pyrosequencing in hybrids from crosses of Arabidopsis accessions differentially expressing this gene. Because expression of both alleles in a hybrid cell is controlled by the same set of regulators, the relative abundance of each allele in the hybrid should be equaled out if trans-regulators cause the differences between accessions. In contrast, if the expression differences between accessions are caused by mutations in cis, the differences in the expression of the alleles in the hybrid will be maintained [39].

We generated F_1_ hybrids by crossing L*er*-1 with Col-0 and with four accessions containing *FRI-wt* alleles (Bor-4, Bur-0, Sha and Knox-10; [14]). In all F_1_ individuals analyzed the relative abundance of each allele measured in genomic DNA was close to 50%, which ensured that the assay had no preference for either of the two alleles (Figure 3c). Analysis of cDNA from the same individuals revealed lower relative abundance of *FRI-Ler* than either the *FRI-wt* or the *FRI-Col* alleles (Figure 3d). These differences suggest that at least part of the phenotypic differences found between accessions carrying the *FRI-Ler* allele and those carrying the *FRI-wt* allele is due to a cis regulatory mutation reducing the expression of *FRI-Ler*. Interestingly, the differences in expression found among accessions classified by their *FRI* allele are larger than the differences observed in allele specific expression in the hybrids (compare Figure 3a and d), suggesting the existence of additional trans-regulators controlling differences in expression among alleles. Further experiments will be required to determine the precise mode of regulation of this gene.

### Effect of coding polymorphisms in *FRI-Ler*

**Figure 4:**
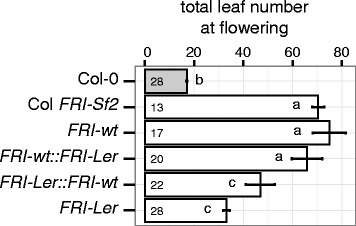
**Flowering time of transgenic lines carrying promoter-swap constructs involving the** ***FRI-Ler*** **and** ***FRI-wt*** **alleles.** Plants were grown in an environmental chamber set to long day conditions and the total number of leaves (rosette + cauline) were counted on the day of the opening of their first flower. Col-0 is the untransformed control and Col *FRI-Sf2* is a near isogenic line containing the *FRI-wt* allele from the accession Sf-2 [2]. Phenotypes were scored in T_1_ individuals for lines carrying the *FRI-wt*, *FRI-wt::FRI-Ler* and *FRI-Ler::FRI-wt* constructs, and in individuals from two independent T_3_ lines for lines carrying the *FRI-Ler* construct (see Material and Methods). Numbers in each bar indicate the number of individual plants analyzed per genotype. Error bars represent the standard error of the mean. Letters in each bar represent significance groups as determined by Tukey HSD test.

In addition to the suggested regulatory polymorphisms causing inhibition of the expression of the *FRI–Ler* allele, its predicted protein differs from the FRI-wt protein in the deletion of 42 amino acids from the N terminal and in one substitution located in a coiled-coil domain (Figure 1d). To test whether these coding differences have an effect on the function of the FRI-L*er* protein compared to FRI-wt, we generated transgenic lines carrying constructs in which the coding region of each allele was placed downstream of the native promoter of the other allele (Figure 4). Due to constraints (see Material and Methods), this experiment was performed using T_1_ lines carrying *FRI-wt* (the *FRI-wt* allele expressed under its native promoter), *FRI-wt::FRI-Ler* (the *FRI-Ler* allele expressed under the *FRI-wt* promoter) or *FRI-Ler::FRI-wt* (the *FRI-wt* allele expressed under the *FRI-Ler* promoter) constructs, and individuals from two independent T_3_ lines carrying the *FRI-Ler* construct (the *FRI-Ler* allele expressed under its native promoter). As expected, lines carrying the wild type promoter driving the expression of the wild type allele (*FRI-wt*) flowered with the same number of leaves as Col *FRI-Sf2*, a line in which the *FRI-wt* allele from the accession Sf-2 was introgressed into Col-0 [2]. In addition, lines carrying constructs with the *FRI-Ler* promoter flowered significantly earlier than those with alleles driven by the *FRI-wt* promoter. This supports our hypothesis that cis-regulatory elements specific to the *FRI-Ler* promoter are the cause of early flowering in accessions carrying this allele. Interestingly, lines carrying the *FRI-Ler* coding region flowered earlier than lines carrying the *FRI-wt* coding region when expressed under the same promoter. Although this acceleration of flowering was not significant, it was observed both when using the *FRI-Ler* promoter and the *FRI-wt* promoter. This result leaves open the possibility that the loss of 42 amino acids and/or the mutation in the coiled coil domain in the *FRI-Ler* protein contribute to accelerating flowering in the accessions that express it.

### Analysis of life history parameters in the *FRI-Col* and *FRI-Ler* accession groups

A major question that derives from the classification of *FRI-Ler* as a semi-functional allele is whether it represents a change in the life history of the accessions that carry it when compared to the accessions carrying the *FRI-Col* allele. Although *FRI-Ler* accessions revealed a significant higher response to vernalization than accessions with the *FRI-Col* allele (Figure 1b), both groups only include early-flowering accessions that are not likely to differ in their general life history strategy. Nevertheless, we investigated the variation in specific life history traits between the two allelic classes, which can result in a distinct ability to adapt to local environments. To address this question, we reanalyzed fitness-related phenotypes for a panel of accessions grown at four different field locations [40]. Although we observed small fitness differences in accessions carrying the *FRI-Col* and *FRI-Ler* alleles at various locations, these were not significant (see Additional file 4). On the other hand, accessions containing the *FRI-wt* allele were often significantly different in fitness traits when compared with *FRI-Ler* and *FRI-Col*. Further experiments will be required to determine if differences between the *FRI-Ler* and *FRI-Col* allelic classes are relevant in specific environmental conditions (e.g. under stress).

## Discussion

*FRI*, an activator of the negative regulator *FLC*, is the major determinant of natural variation in flowering time and vernalization sensitivity in *Arabidopsis thaliana* [3]. So far, natural allelic variants of *FRI* have been clustered into two groups, the functional *FRI-wt* group and the non-functional group that included the *FRI-Col* and the *FRI-Ler* alleles. Here we prove that *FRI-Ler*, the most common allele found so far in summer-annual accessions, is partially functional, although it is a weak allele compared to the *FRI-wt* allele present in winter-annual types. We suggest that the indel found in *FRI-Ler*, including part of the promoter and the beginning of the coding sequence, allows transcription of an active messenger from a downstream methionine. This resulting transcript shows lower expression and perhaps slightly reduced activity than the transcripts from both *FRI-wt* and *FRI-Col*, but has the ability to up regulate *FLC*, delay flowering time and confer vernalization sensitivity. In contrast, the *FRI-Col* allele is unable to confer any of these phenotypes, despite being expressed at a level similar to the *FRI-wt* alleles present in winter-annual accessions.

Although *FRI* has a prominent role, not all variation in *FLC* expression and flowering time observed in Arabidopsis accessions can be explained by variation at this gene. Previous studies have demonstrated that allelic variation at *FLC* itself provides a basis for the development of a summer-annual flowering time habit [24,25,41]. As shown by [15], variation in flowering time may be associated with *FLC* only in the presence of a functional *FRI* allele. Consistent with this, we observed that accessions carrying the *FRI-Ler* or *FRI-wt* alleles, but not those carrying a *FRI-Col* allele, varied considerably in *FLC* expression and flowering time (Figures 1 and 3). Further variation of *FLC* levels can be due to additional factors such as mutations in genes of the autonomous floral promotion pathway [25,42-44]. Moreover, allelic variation at genes directly interacting with *FRI*, namely *SUPPRESSOR OF FRIGIDA 4 (SUF4), FRIGIDA-LIKE 1 (FRL1), FRIGIDA ESSENTIAL 1 (FES1)* and *FLC EXPRESSOR (FLX)*, could have an impact [18]. For instance, it has been shown that L*er* contains a non-functional *FRL1* allele, which is compensated by a functional *FRIGIDA-LIKE 2 (FRL2)* allele [45]. The latter restores *FRI*-mediated up regulation of *FLC* expression and, thus, a late flowering phenotype. In contrast, Col-0 carries a functional *FRL1* allele able to interact with *FRI*, but an impaired *FRL2* allele [45].

The effect on flowering time, *FLC* expression and vernalization response conferred by the *FRI-Ler* allele observed in the present study is not comparable to the effect of the *FRI-wt* allele. Although we show here that both alleles differ greatly in expression (Figure 3), we detect small, non-significant but consistent differences between the *FRI-Ler* and the *FRI-wt* allele driven under the same promoter (Figure 4). This suggests that the putative truncation of the first 42 aa of the N-terminal region and/or the amino acid substitution in FRI-L*er* contribute to the functional difference between alleles (Figure 4). In fact, previous work demonstrated that a deletion of 118 residuals from the FRI N-terminus resulted in reduced functionality. Nevertheless, plants missing that part of the protein still flowered later and revealed higher *FLC* expression levels than both Col-0 and transgenic lines lacking a substantial part of the FRI C-terminus [17]. Further studies used a deletion series to demonstrate that the ability of FRI to act as the scaffold protein for a *FLC* activating complex depends mainly on its C-terminal part [18]. These studies suggest that the truncated FRI-L*er* protein should be able to interact with its known partners to build the FRI-complex and, thus, to induce *FLC* expression. Finally, the amino acid change from leucine in FRI-wt to isoleucine in FRI-L*er* in the first coiled-coil domain is very conservative, but might impair the functionality of the protein (Figure 1d). However, the small differences observed between the *FRI-Ler* and *FRI-wt* alleles expressed under the same promoter suggest that if real, the effect of these mutations on flowering time is minor compared to the effect of their differences in expression.

Despite the functional difference between the two alleles, accessions of the *FRI-Col* and *FRI-Ler* group are all rapid cycling and, thus, do not display a difference in their general life history strategy. Furthermore, differences in fitness traits between both allelic groups could not be detected in the present study. A more comprehensive analysis of larger data sets collected in multiple environments might be required to detect a distinct effect of the *FRI-Ler* allele in nature. In fact, [46] have demonstrated that, depending on the seasonal timing of specific environmental signals, Arabidopsis accessions in nature are capable of both life history strategies. For instance, Col-0 growing during autumn in the field at a specific location in Germany (Halle) displayed a winter-annual habit and flowered almost at the same time as the introgression line Col *FRI-Sf2* that carries a functional *FRI-wt* allele [46]. In contrast, Col *FRI-Sf2* exhibited an unexpected rapid-cycling phenotype when grown in summer at one location in England (Norwich). Nevertheless, L*er* resembled Col-0 with regard to flowering time at all locations tested [46]. Furthermore, we cannot exclude that the *FRI-Ler* allele has a role in the modulation of adaptive parameters other than flowering time and vernalization response. For example, pleiotropic effects of *FRI* have been reported for traits such as water use efficiency and leaf senescence, where the latter is closely linked to plant reproduction [47,48].

## Conclusions

In the present study we disprove the common assumption that the widespread *FRI* allele found in the Arabidopsis L*er* accession is not functional. We demonstrate that the *FRI-Ler* allele increases flowering time and *FLC* expression and induces vernalization responses to levels that are in between the ones observed for the true null *FRI-Col* allele and the fully functional *FRI-wt* allele.

These intermediate phenotypes observed in plants carrying *FRI-Ler* could be explained by the presence of a close to full-length protein never before associated with this allele. We show that the differences in functionality between the *FRI-Ler* and the *FRI-wt* alleles are largely due to expression polymorphisms, although variation in the protein sequence may also play a role.

Using the limited data available from plants grown under natural conditions, we cannot conclude that the *FRI-Ler* allele confers differences in fitness or life history strategies when compared to the *FRI-Col* allele. On the other hand, phenotypic differences between accessions carrying these alleles are clear and could be advantageous under specific natural environments not explored so far.

In summary, we demonstrate the existence of an allelic series in *FRI*. Besides completely functional and non-functional alleles, we have found a widespread allelic class with intermediate functionality. The use of this novel classification will increase the accuracy of adaptive studies in Arabidopsis.

## Methods

### Analysis of published datasets

Leaf number from Recombinant Inbred Lines (RILs) in the L*er* x Col population [31] was analyzed by grouping lines according to their genotypes at the closest molecular markers to *FRI* (m506, chromosome 4 at 0.0 cM) and *FLC* (g4560, chromosome 5 at 17.3 cM). Phenotypic and genotypic data for these individuals was kindly provided by Johan W. van Ooijen and Caroline Dean.

Responses to vernalization from 126 Arabidopsis accessions were obtained from published data [22] and analyzed by subtracting days to flowering with vernalization from days to flowering without vernalization (plants grown in controlled environment rooms with 16 hour light). In this dataset, *FRI* alleles are classified as *FRI-Ler*, *FRI-Col*, non-functional alleles or novel alleles. The rest of the alleles were assumed to be functional (*FRI-wt*) as they do not contain the L*er* or Col-0 indels and the accessions that carry them are late flowering. For our analysis, we considered only those accessions containing alleles classified as *FRI-wt*, *FRI-Ler* or *FRI-Col*. Accessions for which the *FLC* allele was classified as non-functional or novel were removed.

Expression data for *FRI* and *FLC* was obtained from available repositories holding northern hybridization quantification for 137 Arabidopsis accessions (greenhouse grown, 16 h light [38]). Accessions in this dataset were classified according to their *FRI* alleles by combining genotypes from the following sources: [14,21,22,27,37]. The assignment of *FRI* alleles for each accession can be found in Additional file 5.

Fitness data was obtained from Arabidopsis accessions grown in four different field locations [40]. These accessions were classified according to their *FRI* alleles as above.

### Sequencing of *FRI* in Col and L*er*

Genomic DNA of Col-0 and L*er*-1 was extracted from young leaves using a Plant DNeasy Mini Kit (Qiagen, Chatsworth, CA, USA). A region of approximately 3250 bp including the complete *FRI* gene (AT4G00650) and an upstream and downstream region was amplified in overlapping fragments of 600–700 bp using Phusion High-Fidelity DNA Polymerase (New England Biolabs). As a reference, the same region was sequenced for the accession Bil-7 carrying a fully functional *FRI-wt* allele [14]. Pooled PCR products from four independent reactions were purified using QIAquick PCR purification kit (Qiagen, Chatsworth, CA, USA) and sequenced via Sanger sequencing at the Max Planck Genome Centre Cologne. The individual sequences were assembled and aligned against the sequence of Bil-7 using SeqMan Pro (DNAstar, Madison, WI, USA).

### Cloning and phenotyping of transgenics

In order to clone the *FRI-Ler, FRI-wt* and *FRI-Col* alleles, we designed primers flanking positions −1372 to +2691 relative to the *FRI* start codon annotated in TAIR10, which include the complete gene plus an upstream region of 1061 bp and a downstream region of 379 bp. This region was amplified by PCR from DNA of the accessions L*er*-1, Bil-7 and Col-0 using Phusion High-Fidelity DNA Polymerase (New England Biolabs). For *FRI-Ler* and *FRI-Col*, PCR fragments were introduced into the binary vector pCAMBIA2300 making use of EcoRI/BamHI restriction sites. For *FRI-wt*, the PCR fragment was introduced into binary vector pBinGlyRed1 (kindly provided by Ed Cahoon, University of Nebraska) making use of the same EcoRI/BamHI restriction sites.

Promoter-swap constructs were generated using the MultiSite Gateway® Pro 2.0 system (Life Technologies) and genomic DNA from the accessions L*er*-1 (*FRI-Ler*) and Bil-7 (*FRI-wt*). The *FRI-Ler* promoter region was cloned starting from position −1372, relative to the annotated *FRI* start codon in TAIR10, and ending at position +126, which is right before the first ATG downstream of the indel described in *FRI-Ler*. For *FRI-wt,* the promoter region cloned ranged between positions −1372 and −1. Both promoter sequences were introduced into pDONRTM 221 P1-P5r by BP reaction. Similarly, the coding sequences for both alleles together with 379 bp of its downstream sequence (position +1 to position +2691 bp for *FRI-wt* and position +127 to position +2691 for *FRI-Ler*) were cloned and introduced into pDONRTM 221 P5-P2. Finally, *FRI-wt::FRI-Ler* and *FRI-Ler::FRI-wt* constructs were generated from LR reactions using the binary vector pFAST.

All final constructs were transformed into *E. coli* strain Top10 (One Shot® TOP10 chemically competent *E. coli*, Invitrogen). Inserts from positive colonies were sequenced and verified by comparing them to the PCR template sequence or to the expected in silico constructs. Subsequently, all constructs were transformed into *Agrobacterium tumefaciens* strain GV3101 and used for transformation of Col-0 plants by the floral dip method [49].

Transgenic plants carrying *FRI-Ler* or *FRI-Col* constructs (in pCAMBIA) were selected on MS agar plates containing 50 mg/l kanamycin. Independent T_1_ plants carrying a single insertion were identified and homozygous T_3_ plants were used for subsequent analyses. Flowering time in T_3_ lines carrying *FRI-Col* or *FRI-Ler* was measured in the greenhouse under long day conditions (16 h day length) with and without vernalization treatment for four weeks at 4°C.

T_1_ plants carrying the promoter swap between *FRI-Ler* and *FRI-wt* (in pFAST) and those carrying the Bil-7 *FRI-wt* alleles (in pBinGlyRed1) were selected by their red fluorescence under a fluorescence stereomicroscope (Leica MZ16F, Leica Microsystems, Germany) using green light of wavelength about 580 nm. Flowering time was recorded in T_1_ lines carrying the *FRI-wt* allele and the promoter swap lines *FRI-wt:FRI-Ler* and *FRI-Ler::FRI-wt* under long day condition in an environmental chamber. This experiment included plants from two independent T_3_ lines carrying the *FRI-Ler::FRI-Ler* allele (lines 14–1 and 16–4 in Figure 2). Using these T_3_ lines allowed us to sow all the plants in the experiment directly on soil, as *FRI-Ler* T_1_ lines do not have red fluorescent marker and would have required selection on kanamycin plates.

In all experiments, flowering time was quantified as total leaf number (rosette + cauline) on the day the first flower opened.

### Expression analysis using quantitative RT-PCR

For expression analysis of transgenics, seedlings (leaves plus shoot) grown in an environmental chamber at long day conditions (16 h light) were harvested 10 days after sowing. Material of 8 to 10 seedlings was pooled for each of three biological replicates. RNA was extracted using Trizol (Ambion® TRIzol® RNA Isolation Reagent, Life Technologies) and transcribed into cDNA using Super Script® II Reverse Transcriptase (Invitrogen). Quantitative RT-PCR was performed on a CFX384 Touch™ Real-Time PCR Detection System using SYBR Green dye (iQ™ SYBR® Green Supermix, Biorad) using the following gene-specific primers: *FLC*-fwd: 5′-CCGAACTCATGTTGAAGCTTGTTGAG-3′, *FLC*-rev: 5′- CGGAGATTTGTCCAGCAGGTG-3′, *FRI*-fwd: 5′- TGCCTGATCGTGGTAAAGGGAAG-3′ and *FRI*-rev: 5′- AGCACCGGCAATCTCATTCGAAC-3′. Expression values were determined using the standard curve method and normalized to the expression of *PP2A* (*PP2A*-fwd: 5′-TAACGTGGCCAAAATGATGC-3′, *PP2A*-rev: 5′- GTTCTCCACAACCGCTTGGT-3′). Normalized expression was averaged for three biological replicates each analyzed in two or three technical replicates.

### Allele-specific expression analysis

Leaves from F_1_ hybrids involving the L*er*-1 parent were collected at bolting from plants grown in 12 h days in a controlled environmental chamber. Material from F_1_ hybrids involving the Col-0 parent were collected from ten-day old seedlings grown in long days (16 h light) in a controlled environmental chamber. Here, material of 8 to 10 seedlings was pooled per biological replicate. RNA was extracted from 20 mg of frozen material using RNeasy Plant kit (Qiagen) combined with on-column DNase digestion using RNase-Free DNase Set (Qiagen). Subsequently, cDNA was synthesized using SuperScript® III Reverse Transcriptase kit (Invitrogen) in the presence of RNase inhibitor RNasin (Promega) following specifications from the manufacturer. Genomic DNA was extracted from the same samples using DNease Plant kit (Qiagen).

PCR distinguishing *FRI-Ler* versus *FRI-wt* alleles targeted a variant from C (in Bor-4, Bur-0, Sha and Knox-10) to A (in L*er*-1) at chromosome 4, position 269257 (TAIR10). The fragment containing this SNP was amplified using primers 5′Biotin-TCAGTTGCAGTGGAAACATTCA-3′ and 5′-GCGTTTTCGATTGACTCGATGT-3′, and pyrosequencing was performed using primer 5′-TGACTCGATGTGCTTCT-3′. To distinguish the *FRI-Ler* and *FRI-Col* alleles in the L*er* x Col F_1_ hybrid we targeted a variant from A in Col-0 to G in L*er*-1 at chromosome 4, position 269469 (TAIR10). The fragment containing this SNP was amplified using primers 5′Biotin-ATTGTACCGGAGACGTCGAATAA-3′ and 5′-GGCCAATTTCAAAGCTGAAG-3′, and pyrosequencing was performed with primer 5′-CTTTGCTACACATCAACTC-3′. 0.5 μL of cDNA was used for PCR, which was conducted in a solution (2.5 units of Taq polymerase, 2.5 mM MgCl_2_, 200 μM dNTPs, buffer B, 0.2 mM of each dNTP; Bio-Budget) containing 20 pmol of each primer, in the total volume of 25 μL. The PCR products were obtained with 50 cycles (93°C, 45 sec; 60°C, 45 sec; 72°C, 1 min).

Allele-specific mRNA abundances were measured using PyrosequencerAB (Biotage AB, now Qiagen) following manufacturer’s instructions for sample preparation and pyrosequencing reactions. Vacuum sample preparation was performed using 15 μL of PCR product mixed with 5 μL of Streptavidin Sepharose beads (GE Healthcare), 40 μL of PyroMark Binding Buffer (Qiagen) and 20 μL of LiChroSolv water (Merck). Pyrosequencing was performed in a PyroMark Q96 Plate Low (Qiagen) in each well containing 1 μL of sequencing primer (10 μM) and 40 μL of Pyromark Annealing Buffer (Qiagen) using PyroMark Gold Q96 Reagents (Qiagen). Allele-specific expression for each SNP was estimated using the pyrosequencing software (PSQ 96MA 2.1.1, Biotage AB) based on the peak height for each allele at the SNP. A peak correction factor of 0.86 was used for incorporation of dATP αS, as recommended by the manufacturer.

### Availability of supporting data

The coding DNA sequence and translated protein sequence of the *FRI-Ler* allele supporting the results of this article are available through NCBI’s GenBank under accession number KJ545576 (http://www.ncbi.nlm.nih.gov/genbank).

## Additional files

Additional file 1:
**Response to vernalization quantified as the decrease in the total number of leaves at flowering for Arabidopsis accessions grouped by their** ***FRI*** **allele, as described in [** 22 **].**
Additional file 2:
**RNA-seq coverage at the** ***FRI*** **locus in seedlings of seven Arabidopsis accessions carrying the** ***FRI-Ler*** **allele.**
Additional file 3:
**Expression of** ***FRI*** **in transgenic lines carrying the** ***FRI*** **transgene.**
Additional file 4:
**Assessment of fitness differences between accessions containing** ***FRI-Col, FRI-Ler*** **or** ***FRI-wt*** **alleles.**
Additional file 5:
**Assignment of** ***FRI*** **alleles in Arabidopsis accessions used in Figure** 3 **and Additional file** 4 **.** For both articles referenced in the table [38,46], *FRI* alleles are marked with y (yes) when identified in the article and with n (no) when not present in the article.
